# Endocrine Autoimmune Disease as a Fragility of Immune Surveillance against Hypersecreting Mutants

**DOI:** 10.1016/j.immuni.2020.04.022

**Published:** 2020-05-19

**Authors:** Yael Korem Kohanim, Avichai Tendler, Avi Mayo, Nir Friedman, Uri Alon

**Affiliations:** 1 Department of Molecular Cell Biology, Weizmann Institute of Science, Rehovot 76100, Israel; 2 Department of Immunology, Weizmann Institute of Science, Rehovot 76100, Israel

**Keywords:** autoimmune disease, immune surveillance, circuit motif, hypersecreting adenoma, Hashimoto’s thyroiditis, type 1 diabetes, vitiligo, primary biliary cirrhosis, hyperparathyroidism, systems biology

## Abstract

Some endocrine organs are frequent targets of autoimmune attack. Here, we addressed the origin of autoimmune disease from the viewpoint of feedback control. Endocrine tissues maintain mass through feedback loops that balance cell proliferation and removal according to hormone-driven regulatory signals. We hypothesized the existence of a dedicated mechanism that detects and removes mutant cells that missense the signal and therefore hyperproliferate and hypersecrete with potential to disrupt organismal homeostasis. In this mechanism, hypersecreting cells are preferentially eliminated by autoreactive T cells at the cost of a fragility to autoimmune disease. The “autoimmune surveillance of hypersecreting mutants” (ASHM) hypothesis predicts the presence of autoreactive T cells in healthy individuals and the nature of self-antigens as peptides from hormone secretion pathway. It explains why some tissues get prevalent autoimmune disease, whereas others do not and instead show prevalent mutant-expansion disease (e.g., hyperparathyroidism). The ASHM hypothesis is testable, and we discuss experimental follow-up.

## Highlights

• In many endocrine tissues, a signal drives both secretion and proliferation
• Mutant cells that hypersecrete and hyperproliferate thus arise, spoiling homeostasis
• Proposed autoimmune surveillance mechanism eliminates these life-threatening mutants
• Predicts tradeoff between autoimmune disease and hypersecreting adenomas

## Graphical Abstract

**Figure:**
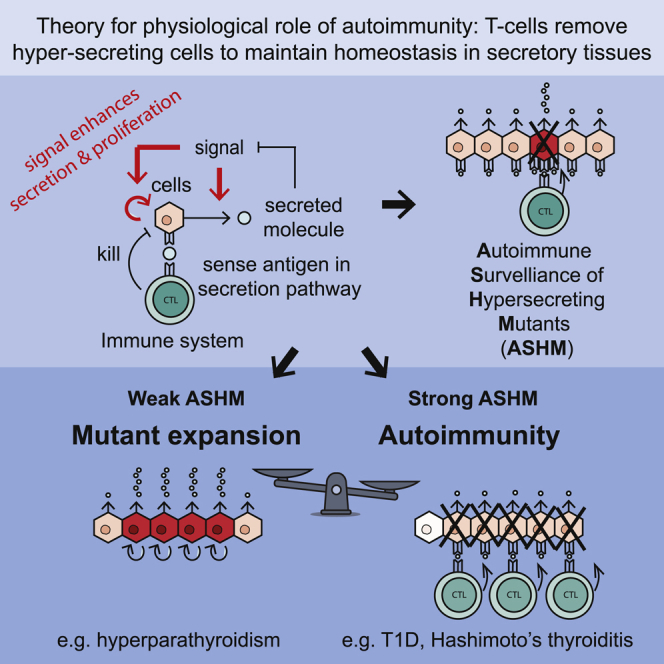

## Teaser

Some endocrine tissues are prone to autoimmune disease, whereas others are not. Korem Kohanim et al. propose the existence of an immune surveillance mechanism wherein auto-reactive T cells remove aberrant hyperproliferating and hypersecreting endocrine cells to prevent disruption of homeostasis. Autoimmunity is the cost of this surveillance mechanism.

## Introduction

**Figure 1:**
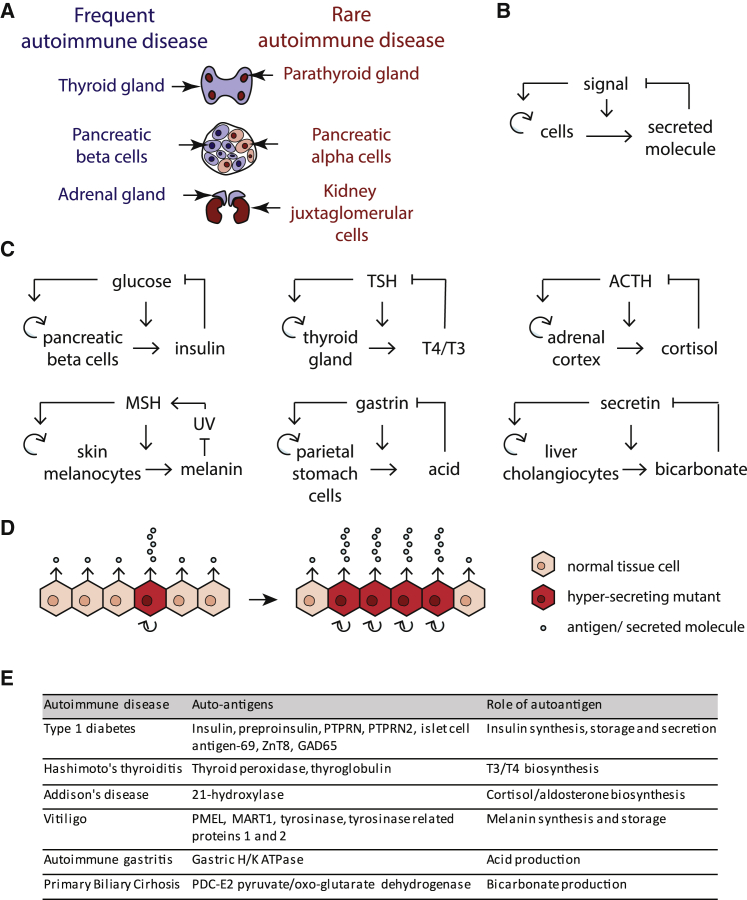
Regulatory Circuits in Secretory Tissues that Show Prevalent Autoimmune Diseases Link HyperSensing of the Input Signal with Hypersecretion and Hyperproliferation (A) Some endocrine tissues are prone to autoimmune diseases, while others are not. (B) The regulatory motif common to all prone tissues. (C) The specific implementation of the motif in each tissue (details in STAR Methods; Table S1). (D) Because of this circuit, mutant cells that hypersense the signal (dark red cells) have a larger growth rate and hypersecrete. Due to their growth advantage, these mutant cells can expand, eventually causing physiological hypersecretion, and hence loss of homeostasis (hyposignal). (E) The autoimmune diseases related to the circuits in (C) have autoantigens in the biosynthesis and secretion pathways of their corresponding secreted factor (Table S1).

Autoimmune diseases can be classified into systemic diseases, such as lupus, rheumatoid arthritis (RA), and systemic sclerosis, and organ specific diseases, such as type 1 diabetes. Many organ-specific diseases occur in endocrine and exocrine organs. In type 1 diabetes, for example, T cells target and kill pancreatic beta cells. Other common T cell-based autoimmune diseases include Hashimoto’s disease of the thyroid, Addison’s disease of the adrenal cortex, and vitiligo, wherein skin melanocytes are the targets of autoimmune attack. These autoimmune diseases have a relatively young age of onset, and their prevalence typically ranges between 0.1%–1% of the population (Cooper et al., 2009). Interestingly, some endocrine organs are very rarely targets of autoimmunity, including the parathyroid and pancreatic alpha cells, for reasons that are not understood (Figure 1A).

The fundamental origins of autoimmune disease are unknown. One point of view is that the T cells that attack specific organs are escapees of protection mechanisms that act to prevent autoimmunity. These protection mechanisms include induction of central tolerance in the thymus and peripheral tolerance (Xing and Hogquist, 2012). Pruning of self-reactive T cells in the thymus not only involves clonal deletion of T cells with high affinity to self-antigens, but also the generation of CD4^+^ FOXP3-dependent Treg cells from some self-reactive clones (Stritesky et al., 2012). These Treg cells suppress the activity of self-reactive T cell clones, contributing to peripheral tolerance. Indeed, genetic predisposition to autoimmune disease lies primarily in genes related to major histocompatibility complex (MHC) class II genes, as well as T cell selection in the thymus and Treg cell development or function (Hewagama and Richardson, 2009, Ray and Hacohen, 2015). Other protective mechanisms include various checkpoints of T cell activation in the immune periphery, evidenced by the occurrence of auto-immune diseases in patients receiving immune checkpoint blockade therapy (Caspi, 2008).

Multiple mechanisms can contribute to the generation of antigens that promote autoimmunity. These include post-translational modifications (PTM) of autoantigens, such as deamidation of pro-insulin in T1D, and citrullination of autoantigens in RA (Doyle and Mamula, 2001). Furthermore, misfolded proteins can be presented by MHC class II and thus seen as non-self by T cells (Arase et al., 2017). Translational errors giving rise to defective ribosomal products (DRiPs) can also act as self-epitopes in autoimmune disease (Kracht et al., 2017). Certain autoantigens may be more likely to be presented by specific MHC class I molecules and more susceptible to PTM associated with environmental signals. Another risk factor is infection. One theory is that pathogens have proteins that resemble certain self-proteins, setting off autoimmune attack through cross-reactivity (Cusick et al., 2012), or that infections release antigen and produce local inflammation and thereby increase chances of breakdown of tolerance mechanisms (Hafler, 1999).

A different point of view is that the self-reactive T cells play an important physiological role (Cohen, 2004, Kracht et al., 2016, Richards et al., 2016, Schwartz and Cohen, 2000, Schwartz and Raposo, 2014). This is supported by evidence that self-reactive T cells are found in healthy individuals, including T cells that recognize beta cells, melanocytes, and thyroid cells (Culina et al., 2018, Hogquist and Jameson, 2014, Liu et al., 2015, Madi et al., 2014, Ota et al., 1990, Semana et al., 1999, Yu et al., 2015). The young age of onset and devastating nature of organ-specific autoimmune diseases carries a large evolutionary cost. Thus, their relatively high prevalence may suggest that they originate from some process that is essential for human biology. What this process exactly is, however, remains unknown. It would be important to identify an essential biological process that requires self-reactive T cells toward specific antigens in specific endocrine glands. Such a process would supply the healthy counterpart to autoimmune diseases.

Here, we propose that some autoreactive T cells remove hypersecreting mutant cells that can over-proliferate and threaten homeostasis. We test several predictions of this theory: (1) that relevant autoantigens are derived from proteins central to the production of the secreted molecule and (2) that endocrine tissues that are rarely autoimmune targets are instead prone to mutant-clone expansion diseases with hypersecretion. Using mathematical modeling, we outline the regulatory circuits via which this proposed autoimmune surveillance mechanism might work. The hypothesis presented here is readily testable and falsifiable, and we discuss possible experimental tests.

## Results

### Secretory Organs Prone to Autoimmune Disease Share a Common Feedback Circuit Motif

We focused on autoimmune diseases targeting endocrine and exocrine organs. Table S1 lists organ-specific autoimmune diseases of high prevalence (more than 0.1% lifetime prevalence). In these diseases, T cells attack pancreatic beta cells, thyroid, adrenal cortex, melanocytes, gastric parietal cells, and liver cholangiocytes (Figure 1A).

We considered these systems from the point of view of tissue-level regulatory circuits. All of these tissues have cell turnover, in which cells proliferate and are removed on a timescale of months. As shown by Karin et al., (2016), in order to maintain organ size, cell proliferation and removal must be precisely balanced and hence must be controlled by a feedback loop. This feedback loop prevents exponential growth or collapse of the tissue. In order to provide organ functional mass that matches physiological needs, the feedback must operate based on input signals that are related to the tissue function.

We therefore sought in each organ system what signals control cell proliferation and hormone secretion. We recognized that these organs share a regulatory motif: the input signal (e.g., blood glucose in the case of beta cells) increases both the secretion of a hormone (e.g., insulin) and the growth rate of the secreting cells by increasing proliferation and/or reducing apoptosis. The secreted factor, in turn, regulates the input signal (e.g., insulin reduces blood glucose) (Figures 1B and 1C). Thus, secretion and proliferation are linked in these cells. If there is too little hormone, signal level increases and the circuit makes the cells secrete more hormone, and also proliferate to enable larger hormone supply over longer timescales.

Analyses of these circuits revealed a shared fragility to missensing mutant cells (Figure 1D). A mis-sensing mutant is defined as a cell with a mutation in the sensing pathway that causes it to over-respond to the signal. As a result, the mutant cell both hyperproliferates and hypersecretes. Examples occur in clinical contexts: mutations in glucokinase cause beta cells to over-sense glucose and as a result to hypersecrete insulin (Glaser et al., 1998, James et al., 2009, Matschinsky, 2002), causing hypoglycemia. In the thyroid, thyroid stimulating hormone (TSH)-receptor mutants grow into nodules that hyper-secrete thyroid hormone (Holzapfel et al., 2002), causing hyperthyroidism. Over-sensing in principle might be not only due to a genetic change, but also due to a long-lasting epigenetic aberration that is passed on to daughter cells. This will generate cell-cell variation in hormone expression; such variation is seen, for example, in single cell measurements of insulin expression in beta cells (Farack et al., 2019).

Because missensing mutant cells would have a higher growth rate than other cells in the organ, they could compete with other cells and can expand to form clones. When these mutant clones reach a large enough fraction of the total cell population of the relevant organ, hypersecretion begins to interfere with organismal homeostasis.

At first sight, it might seem that such mutations arise rarely. However, when considering the mutational target and mutation rates, it is easy to see that these mutations are practically unavoidable. An organ like the thyroid weighs about 10 g and contains about 10^10^ thyroid-hormone-secreting cells. Therefore, to develop this organ requires at least 10^10^ cell divisions. The human mutation rate is about p = 10^−9^/bp/division (Milholland et al., 2017). Thus, any given mutation will arise about ten times in the thyroid by the time it has fully developed. There are at least 45 different activating mutations in the TSH receptor that give rise to thyroid hypersecreting nodules (Iosco and Rhoden, 2011). Thus, the thyroid is expected to have hundreds of hypersecreting hyperproliferating nodules in every individual. This would cause toxic hyperthyroidism (Franklyn and Boelaert, 2012). Similarly, there are about 10^9^ beta cells in humans, and tens of mutations can give rise to hypersecreting adenomas (Arnoux et al., 2011), so that potentially lethal hypoglycemia due to insulin-secreting adenomas would seem to be inevitable. Additional mutations likely arise in the adult as a result of tissue turnover, especially when the gland function is strained, such as in low iodine conditions, which cause thyroid growth (Dumont et al., 1992), or during pregnancy, which involves proliferation in several endocrine glands.

Thus, the expansion of mutant cells within organs sharing this regulatory motif would present an almost unavoidable threat to systemic homeostasis. There is therefore scope for a system that would regulate that number of mutant cells in these organs; this system may itself carry fragilities.

Based on these analyses, we propose the existence of a system based on autoimmunity that restricts mutant cells, which we call *autoimmune surveillance of hypersecreting mutants*, ASHM. In ASHM, the T cells would detect and remove cells with high expression of proteins in the secretion pathway relative to neighboring cells. In this way, T cells targeting specific types of self-antigens restrict the growth of hypersecreting cells relative to other cells in the organ.

ASHM makes the following predictions: (1) the autoantigens must be in the pathway making the secreted molecule, to allow for specific targeting of hypersecreting cells; (2) endocrine tissues with rare autoimmune disease (weak ASHM) should show common expansion of hypersecreting mutants; (3) Autoreactive T cells that target autoantigens related to secreted molecules exist in healthy individuals.

### Autoantigens in Endocrine Autoimmune Diseases Are Often in the Secretion Pathway

To test the prediction that the autoantigens must be in the pathway making the secreted molecule, we reviewed the literature for characteristics of major antigens in the endocrine and exocrine autoimmune diseases (Figure 1E; Table S1). All but one of the known antigens associated with these autoimmune diseases are derived from proteins within pathways that generate secreted molecules. The major autoantigens associated with T1D, preproinsulin and protein tyrosine phosphatase, are both in the insulin synthesis pathways. In Addison’s disease, the major autoantigen is derived from 21-hydroxylase, an enzyme that performs a rate limiting step in cortisol and aldosterone synthesis. Autoantigens associated with Hashimoto’s thyroiditis are derived from thyroglobulin and TPO, critical proteins within thyroxine (T4) synthesis. In vitiligo, the autoantigen derives from an enzyme performing a rate limiting step in melanin production, and the main autoantigen associated with autoimmune gastritis derives from the acid transporter. Our review identified one autoantigen associated with autoimmunity that is not related to the biosynthesis of the secreted molecule, namely GAD65 in T1D. This case may require additional explanation, such as epitope spreading, as has been suggested for this antigen (Codina-Busqueta et al., 2011).

These antigens are specific to their cell type. It is thus instructive to consider also a case in which the autoantigen is present in many different cell types: the case of primary biliary cirrhosis (PBC). PBC is an autoimmune disease in which T cells attack liver cholangiocytes. The autoantigen is a mitochondrial protein (anti-mitochondrial antigen, or AMA). It is puzzling that a mitochondrial antigen, present in all tissues, would lead to attack on specific cells, the liver cholangiocytes (Lleo et al., 2014). We considered this in light of the proposed ASHM concept. Cholangiocytes produce bicarbonate for bile secretion (Figure 1C). According to the ASHM hypothesis, the antigen should be in the bicarbonate secretion pathway. Indeed, the autoantigen is a regulatory subunit of pyruvate-dehydrogenase and oxo-glutarate dehydrogenase. These dehydrogenases are the main producers of CO_2_ in the cell, through glycolysis and the TCA cycle. CO_2_ is the basis for production of bicarbonate. Thus, cholangiocyte mutant cells that hypersecrete bicarbonate are likely to present higher amounts of AMA antigen than their neighbors. These mutant cells can be removed by ASHM, without affecting other tissues in which there is no growth-advantage for cells that highly express this antigen. Thus, the ASHM hypothesis offers a functional explanation for the mitochondrial antigen in PBC.

### Endocrine Tissues that Rarely Exhibit Autoimmune Disease Are Prone to Mutant-Expansion Diseases

**Figure 2:**
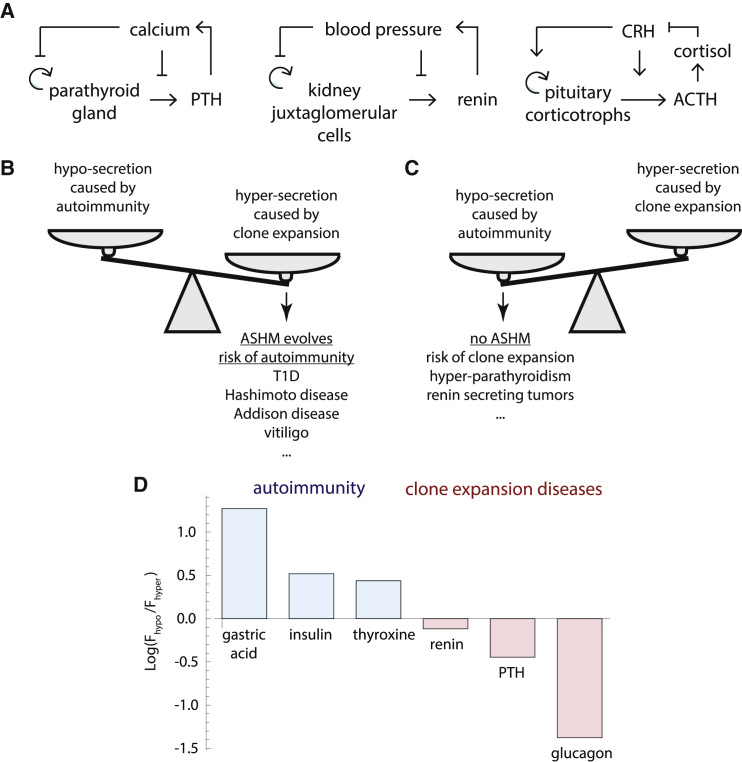
Circuits for Three Endocrine Tissues that Very Rarely Show Autoimmune Disease (A) Circuits for the parathyroid, renin, and pituitary systems (details in STAR Methods; Table S2). (B) When the cost of hypersecretion exceeds the cost of autoimmunity, ASHM can be selected. (C) Otherwise, ASHM is not selected and diseases of hypersecreting clone expansion are expected. (D) A measure C for cost of hypersecretion versus equivalent hyposecretion for six endocrine and exocrine systems. Blue, systems with prevalent autoimmune disease; red, systems with rare autoimmune disease. For molecule x, C is F_hypo_ (F_hyper_) where F_hypo_ (F_hyper_) is the ratio of the normal concentration of x to the lethal level at low (high) perturbations (STAR Methods).

The second major prediction of the ASHM hypothesis is that secretory organs that lack prevalent autoimmune disease should be prone to non-transient expansion of hypersecreting mutant cells. These organs will therefore show prevalent diseases of hypersecretion. To test this, we considered endocrine systems that very rarely get autoimmune diseases and for which cell growth regulation is well studied (Figure 2A; Table S2). These include the parathyroid gland, the pituitary gland, renin-secreting cells in the kidney, and alpha cells in the pancreas.

In some of these systems, the feedback circuit is different: the input signal inhibits proliferation and secretion, instead of activating these processes as in Figure 2. The circuit is still prone to missensing mutant expansion. Such expansion causes hyperregulation of the input signal, as opposed to hyporegulation in the circuits of Figure 1.

We hypothesized that these systems rarely exhibit autoimmune disease because there is no ASHM of these tissues—or a weak version of it. This predicts that such tissues will show relatively frequent expansion of mutant clones that hypersecrete.

Review of the literature revealed that such expansion occurs frequently in the case of the parathyroid gland. Mutant cells that under-sense calcium proliferate, and cause hypercalcemia, in a disease called primary hyperparathyroidism. This disease is prevalent at old age, in as many as 1 of 50 postmenopausal women (Adami et al., 2002). The pituitary gland is also a rare target of autoimmune disease, with about 500 cases reported in 30 years (Caturegli et al., 2008). However, mutant cell expansion is common in the pituitary, estimated at about 20% of the population (Ezzat et al., 2004) (Table S2). The chance per cell for an ACTH-secreting pituitary adenoma is about 250-fold greater than for a cortisol-secreting adenoma in the much larger adrenal cortex (STAR Methods). Such hypersecreting adenomas lead to endocrine problems: Cushing’s disease when ACTH is hypersecreted, acromegaly when growth hormone is hypersecreted, hypergonadism when sex hormones are hypersecreted and hyperthyroidism when TSH is hypersecreted. Clone expansion disease also occurs in the renin system in the kidney, called renin-secreting juxtaglomerular cell tumors, or reninomas. These rare tumors hypersecrete renin and cause high blood pressure in adolescents and young adults. (Haab et al., 1995, Kuroda et al., 2011) (Table S2).

Thus, endocrine tissues that are seldom targets of autoimmune disease exhibit prevalent mutant hypersecretion diseases. We predict that a similar mutant-expansion disease might occur in alpha cells, whose regulatory circuit is more complicated (STAR Methods), resulting in hyperglucagonism and hyperglycemia. Such expansion phenomena have been reported, although other explanations have been offered (Feng et al., 2017, Liu et al., 2011, Unger and Cherrington, 2012).

One may speculate about a principle determining whether a tissue will be an autoimmune target versus being susceptible to clone-expansion disease: if the fitness cost of hypersecretion or hyperexpansion is higher than the fitness cost of autoimmunity, ASHM will evolve (Figure 2B) (Stearns and Medzhitov, 2016). ASHM of these tissues would prevent disease in many individuals, at the cost of young-onset autoimmune diseases in a few. For example, the fitness cost of hyperinsulinemia is very large because hypoglycaemia is lethal. ASHM prevents this lethality, at the cost of T1D. Conversely, if the fitness cost of hypersecretion or hyperexpansion is lower than the fitness cost of autoimmunity, ASHM will not evolve or will be weak (Figure 2C). These systems will instead exhibit mutant-takeover diseases of hypersecretion, whose prevalence rises with age. For example, the fitness cost of parathyroid mutant takeover is tolerable (mild hypercalcemia leading to gradual bone damage and cognitive effects), as compared with acutely lethal hypocalcemia that could result from an auto-immune disease of the parathyroid.

Testing this principle requires knowing the fitness costs of hyper- and hyposecretion, which are difficult to estimate. As a first approximation, we sought a proxy for the relative fitness costs by estimating the deviation from normal ranges in which lethality occurs. For example, lethal hypocalcemia occurs when free calcium drops by a factor of about F_hypo_ = 1.3 (normal range is 1.1–1.3 mM, and lethality occurs below 0.9 mM). Lethal hypercalcemia occurs when F_hyper_ = 2–2.5 (above 2.5–3 mM). No ASHM is predicted in this case because F_hypo_ is much smaller than F_hyper_. Conversely, we reasoned that ASHM would be favored if F_hyper_ is smaller than F_hypo_, because mutant-expansion leading to hypersecretion would be dangerous to organismal health as compared with a similar level of hyposecretion caused by autoimmune surveillance. For insulin-mediated regulation of glucose levels, for example, lethality occurs below 3 mM blood glucose, which is 1.7-fold below the normal 5 mM, whereas a clinically abnormal high level (risk of ketoacidosis) is estimated at 2.8-fold above normal.

We found estimates for six of the relevant factors in this study (Figure 2D; STAR Methods). For gastric acid, insulin (glucose), and thyroxin, F_hyper_ is smaller than F_hypo_, consistent with autoimmune disease. In contrast, for calcium (PTH), blood pressure (renin) and glucagon, the opposite is true, consistent with mutant-expansion diseases. These rudimentary estimates support the proposal that autoimmune disease is likely in tissues where hypersecretion carries a high fitness cost, whereas mutant-expansion diseases is likely in tissues where hyposecretion carries a higher cost.

Additional factors may include the probability for a missensing mutant, which is smaller in small tissues of under 0.1 g, such as pituitary and parathyroid (and hence less need for ASHM), compared with larger tissues on the order of 10 g, such as the thyroid and adrenal cortex (and hence more need for ASHM).

Organs that rarely exhibit autoimmune disease, such as the parathyroid and pituitary, can nevertheless be subject to autoimmune attack in certain situations: immune checkpoint therapy or congenital mutations (e.g., in AIRE) leading to polyglandular autoimmune disease. This suggests that these organs may have a reduced amount of ASHM rather than no ASHM at all. Possible mechanisms for reduced ASHM include greater expression of the relevant tissue proteins in the thymus under AIRE control, trimming potentially reactive T cells from the repertoire, or increasing the level of tissue-specific Treg cells. An alternative mechanism is reduced antigen presentation in these tissues. These possibilities are experimentally testable.

### The Public T Cell Repertoire Includes TCRs that recognize Auto-Antigens Involved in Endocrine Autoimmune Disease

We next considered the plausibility of ASHM in terms of what T cells can do. ASHM requires the immune effector cells to differentially recognize and remove cells that make more autoantigens than their neighbors. Such differential killing is unlikely if the antigen recognition is very strong. For strong antigens, such as certain foreign antigens, a single or a few peptide-MHC (pMHC) complexes per cell have been shown to activate T cells (Huang et al., 2013). However, for lower affinity recognition there is evidence for differential sensitivity based on the number of MHC molecules presenting the antigen on the cell surface (Aleksic et al., 2010, Chervin et al., 2009, Gadhamsetty et al., 2017, Henrickson et al., 2008, Holler and Kranz, 2003, Porgador et al., 1997, Tischer and Weiner, 2019, Voisinne et al., 2015). For example, flow cytometry experiments on CD8 T cells indicate that mid-range affinity antigens have a cooperative activation curve with Hill coefficients of n = 3–5 (Lever et al., 2016). Such cooperativity was suggested to stem from T cell receptor (TCR) kinetic proof reading mechanisms (Lever et al., 2016), inter-TCR interactions within a TCR nanocluster (Martin-Blanco et al., 2018), and/or temporal T cell-target interaction dynamics (Aleksic et al., 2010, Halle et al., 2016).

Indeed, T cells with very strong autoantigen binding are trimmed from the emerging repertoire in the thymus, based on low-level expression of peripheral organ-specific proteins in the thymus (Hogquist and Jameson, 2014). This is consistent with the notion that the TCR repertoire is adjusted for recognition selectivity where a quantitative difference in protein expression of 2-to-3-fold would provide sufficient discrimination to achieve the postulated detection of hypersecretors. We conclude that T cells may be capable of differential killing according to autoantigen levels required for ASHM, especially if the recognition is moderate.

Because ASHM is predicted to be common to all individuals, a further prediction is that TCRs that are responsible for organ-specific autoimmune diseases and are expanded in individuals with these diseases should also be present in all healthy individuals, namely in the public T cell repertoire. To test this, we sought TCR sequences derived from patients or mouse models of the diseases, and which interact with the autoantigens (Table S4). Such TCR beta-chain sequences were identified for T1D (proinsulin, ZNT8, GAD65, and islet autoreactive TCRs) (Culina et al., 2018, Gomez-Tourino et al., 2017), Hashimoto’s thyroiditis (thyroglobulin) (Matsuoka et al., 1994), and vitiligo (PMEL, MART1) (Lang et al., 2001, Zarour et al., 1996).

We compared these sequences with databases of public T cell receptor sequences that are shared in healthy humans or healthy mice (Emerson et al., 2017, Madi et al., 2014; Table S5). We found 30 perfect matches for TCRs from T1D, Hashimoto’s, and vitiligo (Table S3, STAR Methods). Four of these perfect matches occur in both the mouse and human datasets in a set of 92 TCR sequences common to both organisms (underlined in Table S3). We conclude that the TCRs that participate in the disease process are also found in the public TCR repertoire, consistent with their postulated physiological role in ASHM.

### Mathematical Modeling Suggests that ASHM Can Eliminate Mutant Cells while Killing Few Normal Cells

Finally, we use mathematical modeling in order to ask what features of ASHM are essential in order to protect from hypersecreting mutants. The conclusion of this analysis is that ASHM can protect against missensing mutants even when it kills at a rate that is smaller than the natural turnover of the tissue. For ASHM to work effectively, it needs to attack cells at a rate that is strongly nonlinear in antigen level (cooperative response). However, too high killing rates lead to tissue loss and autoimmune disease, illustrating the need for tight regulation of ASHM.

**Figure 3:**
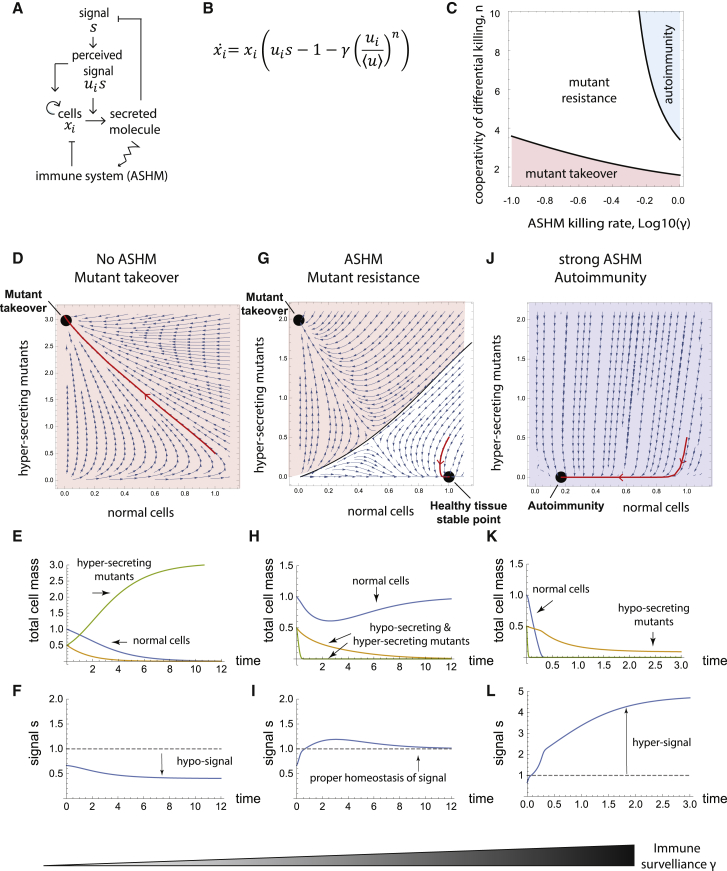
Mathematical Model Indicates that ASHM Can Protect Against Missensing Mutants with Cooperative Killing that Is Infrequent Compared with Natural Turnover (A) Scheme for ASHM mechanism. (B) Equation for the ASHM model: clones of cells $x_{i}$ with missensing distortion $u_{i}$ are subject to ASHM with killing rate γand cooperativity n (STAR Methods). Tregs inhibit killing in proportion to the average antigen presentation from the tissue, normalizing $u_{i}\;$by its tissue mean $<u>=\sum u_{i}x_{i}/\sum x_{i}$ (STAR Methods). (C) Mutant takeover (shown for *u* = 2) occurs when ASHM is not cooperative (low n), autoimmunity (killing of >50% of cells) occurs when ASHM is too strong (high γ). A parameter range (white) allows for removal of mis-sensing mutants without sizable loss of tissue. (D–L) Low γ(γ=0.01) (D–F) showing hypersecreting mutant (u = 2) takeover and hyporegulation of signal, (G–I) γ=0.25 showing mutant elimination and normo-regulation unless mutant population is large, (J–L) strong ASHM (γ=2) showing loss of tissue and hyperregulation of signal, with hyposensing mutant takeover (u = 0.5). Phase plane analysis (D, G, and J) shows hypersecreting mutant and wild-type populations (*u* = 2, *u* = 1 respectively) (STAR Methods). Black dots, stable fixed points. Population dynamics simulations (E, H,and K) show competition between hyper-secreting mutants (green), hypo-secreting mutants (orange) and wild-type cells (blue). (F, I,and L)—signal dynamics.

To model ASHM, we consider the growth rate of a cell clone which senses the input signal *s* (STAR Methods). Mutant cells sense a distorted input signal, which we call the “perceived signal”, so that they sense *u* times more signal than wild-type cells. The perceived signal determines the growth rate and secretion rate of the cells. Cells are also removed by ASHM, which kills cells at a rate that is a function of their antigen level, which in turn is proportional to their secretion rate (Figure 3A). ASHM killing is cooperative as described by a Hill function with coefficient *n*. As mentioned above, experiments suggest high cooperativity. The killing rate of the ASHM system is described by the parameter γ, which denotes the rate of cell deaths in healthy tissue caused by surveillance relative to natural turnover.

Since cytotoxic T cell function is inhibited by Treg cells, we used a minimal description of Treg cell effects (STAR Methods). Treg cells are activated by antigens collected by antigen-presenting cells from the tissue. We thus model the effect of Treg cells by suppressing killing according to the average level of antigen in the tissue. Mathematically, this makes ASHM killing sensitive to antigen presentation by each target cell *relative to the tissue mean*. A similar immune response to relative change in antigen has been theoretically proposed by Sontag (Sontag, 2017), based on earlier studies (Arias et al., 2015, Grossman and Paul, 1992). Other tolerance mechanisms, such as checkpoint inhibition of T cell action, might provide similar effects.

The ASHM mechanism can remove mutants but avoid autoimmunity for a wide range of model parameters (Figure 3C). Physiological changes that increase secretion in the entire tissue, such as increased demand for the hormone, do not lead to increased steady-state immune killing, likely because of a proportional increase in Treg cells.

We further asked whether an ASHM mechanism can remove *all* possible missensing mutants (all values of the perceived-signal parameter *u*). In other words, we asked whether ASHM can provide an “evolutionary stable strategy” (Smith and Price, 1973) in which no mis-sensing mutant can invade and expand in a wild-type population of cells (STAR Methods). We find that ASHM can indeed remove all possible missensing mutants provided that the ASHM killing rate γ equals 1/(*n*-1) times the natural cell removal-rate in the tissue, where *n* is the Hill cooperativity of immune discrimination. Given the high observed cooperativity of cytotoxic T cells, ASHM can work with a small killing rate relative to the natural cell removal rate (γ<0.5).

To illustrate the dynamics of ASHM, in Figure 3 we plot the dynamics of a hypersensing mutant clone that senses twice the true signal (*u = 2*) (STAR Methods). Without ASHM, a mutant cell in a wild-type tissue expands exponentially and takes over (Figures 3D and 3E). As a result, homeostasis is lost (Figure 3F). The same mutant cell vanishes with ASHM (Figures 3G–3I). If the mutant population reaches a critical size, however, ASHM can no longer control it, and the mutant takes over the tissue, as shown in a phase portrait in Figure 3G (orange region). Thus, ASHM is a frequency-dependent mechanism: if all cells are mutant, ASHM cannot distinguish between them and selectively eliminate mutants. Thus, ASHM cannot help in cases of germline missensing mutations. One can prove that ASHM works for a general class of feedback models (STAR Methods).

**Figure 4:**
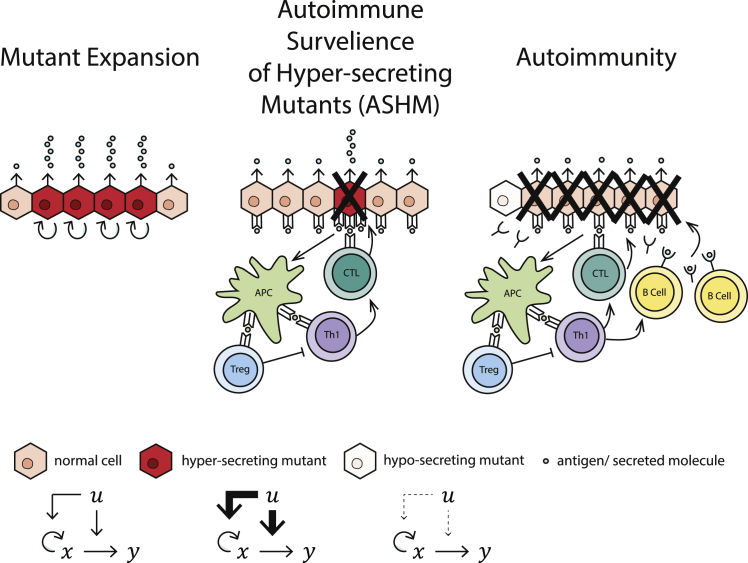
The ASHM Mechanism Can Prevent Hypersecreting Clone Expansion—at the Cost of Risk of Autoimmune Disease ASHM T cells recognize antigens in the secretion pathway to selectively eliminate cells that secrete more than their neighbors. When overactivated, the same cells can set off a persistent immune response including B cell activation that kills much of the tissue, except for hyposecreting clones.

Autoimmune disease can be modeled by raising the killing rate γ (Figures 3J–3L). This represents a situation where memory T cells are activated leading to sustained immune attack and memory (Figure 4). The model predicts that in advanced autoimmune disease, the tissue will not be wholly destroyed but will consist of hyposecreting cells (Figure 3K). These are variants or mutants that proliferate slowly and secrete poorly, as has been observed in T1D. (Keenan et al., 2010, Liu et al., 2009, Rui et al., 2017). They evade immune attack because of the ratiometric killing in the model and can persist as a small population. The larger the immune killing rate γ, the smaller this residual population.

## Discussion

We presented the hypothesis that endocrine organ-specific autoimmune diseases originate from an essential physiological mechanism that removes deleterious hypersecreting mutants. We find that endocrine and exocrine glands prone to autoimmune disease share a regulatory motif in which the signal that they control increases both cell proliferation and secretion. Such feedback gives mutant cells that missense the signal a growth advantage, leading to clone expansion that can ruin homeostasis. To remove these mutants in cases where hypersecretion is life-threatening, we propose an ASHM mechanism. We find evidence for the basic requirements of this mechanism: the major autoantigens in the autoimmune disease are from proteins in the secretion pathways, T cells against these autoantigens are in the public T cell repertoire shared by most healthy individuals, and immune killing can differentiate hypersecreting cells from other cells. This picture predicts that endocrine tissues that rarely get autoimmune disease are instead prone to hypersecreting mutant expansion, explaining the prevalence of hyperparathyroidism, hypersecreting pituitary adenomas, and several other hormone-secreting malignancies (Table S2). We show mathematically that ASHM can protect against missensing mutants with a relatively low killing rate compared to the natural tissue turnover.

Together with its essential mutant-removal function, ASHM poses a risk because it can degrade into autoimmune disease. The outbreak of autoimmune disease has a stochastic element, which is exemplified by homozygous twins that show only 20%–60% concordance (Hewagama and Richardson, 2009). Onset of autoimmune disease can occur, for example, if there is a change in the tissue that increases antigen presentation (Khailaie et al., 2013, Pradeu et al., 2013, Sontag, 2017). Possibilities include tissue damage that causes release of antigen, cell damage that leads to senescence (Thompson et al., 2019), or cell overload that leads to expression of strongly immunogenic variants of the antigen (Kracht et al., 2016, Kracht et al., 2017). These changes might lead to immune response as if the autoantigen had a microbial origin. MHC class II genetic variants are among the strongest genetic determinants of susceptibility to autoimmune disease: these variants may cause T cells to be activated at differing levels of the specific peptides for ASHM, thus affecting thymic selection and/or the thresholds for activating effector cells or Treg cells, and hence modifying the probability of disease.

ASHM is presumably tightly regulated by Treg cells and immune checkpoints and held at an intermediate activity that avoids killing too many healthy cells and, at the same time, eliminates hypersecretors. Thus, weakening immune checkpoints as in cancer immune therapy commonly leads to endocrine autoimmune diseases (Caspi, 2008). Similarly, mutations in genes involved in immune checkpoints or Treg cell development and function are known to increase the risk for autoimmunity (Hewagama and Richardson, 2009).

In addition to ASHM, tissues with the present regulatory motif often have another mechanism against missensing mutants. This mechanism is cell-autonomous biphasic control, in which the signal promotes growth of cells at low levels but kills cells at very high signal levels (Karin and Alon, 2017). For example, glucose drives the growth of beta cells at low-to-medium concentrations but drives their death through glucotoxicity at high concentrations. Similarly, calcium has a biphasic effect on the parathyroid cells that control it. This biphasic mechanism is useful to prevent extreme hypersensing mutants: these mutants perceive high levels of signal and kill themselves. However, biphasic control is vulnerable to mild mutants, which missense the signal at below its toxic levels; removing such mild mutants require an additional mechanism such as ASHM. This observation is in line with the mild nature of the missensing mutants found in the expanding clones of hyperparathyroidism (Yano et al., 2000). Biphasic control may not be feasible in tissues that need to sense the signal over a wide dynamic range, such as the TSH signals to the thyroid which can span three orders of concentrations (Dietrich et al., 2012, Hoermann et al., 2016).

The ASHM model predicts that self-reactive T cells against the specific autoimmune antigens will be found in healthy people. Several studies support this prediction (Cao et al., 2015, Liu et al., 2015, Madi et al., 2014, Semana et al., 1999, Wucherpfennig et al., 1994, Yu et al., 2015). For example, Culina et al. found fully functional cytotoxic islet-reactive CD8+ T cells in the blood of healthy people, in amounts similar to people with T1D (in T1D there are more of these cells in the pancreas compared to healthy people) (Culina et al., 2018). Here, we further find that T cell receptor beta-chains against specific disease antigens are found in the public T cell repertoire shared by healthy individuals.

The T cells involved in ASHM may be low-to-mid affinity CD8+ T cells that escape thymic pruning. These do not overcome inhibitory signals if the MHC class I-peptide levels on the surface are normal, and thus do not precipitate autoimmunity. However, they may cross the activation threshold and overcome inhibitory regulation once the level is high in hypersecreting cells and eliminate these aberrant cells. An interplay of inhibitory and costimulatory signals is probably involved. In our model, T cells can kill at a low rate compared with normal tissue turnover and still give the desired effect of giving hypersecreting mutants a selective disadvantage, as long as the T cells are biased toward removing cells with more antigen than their neighbors.

The ASHM mechanism is in line with the view that self-reactive immune cells play a role in tissue homeostasis and body maintenance (Cohen, 2004, Kracht et al., 2016, Schwartz and Cohen, 2000, Schwartz and Raposo, 2014). It proposes that self-reactive T cells have an essential role in removing hypersecreting mutants and are not merely unwanted escapees of peripheral clonal deletion or anergy or due to errors of elimination in the thymus. Along these lines, another role of the proposed ASHM mechanism could be to trim away hormone-secreting cancers or to remove cells that hypersecrete for non-genomic reasons, such as stochastic fluctuations in receptor number or other parts of the sensory or secretory apparatus. Such a trimming role is especially relevant in tissues with slow turnover in which hypersecreting cells could have a deleterious local effect for prolonged periods.

The ASHM mechanism can be experimentally tested. A critical test would be to examine the consequences of a systemic lack of T cells, which is predicted to enhance the prevalence of hypersecreting mutant expansion in endocrine organs. Possible experimental systems include immune-deficient mice, although the small cell numbers in mice predicts much rarer occurrence of hypersecreting mutants compared with humans. Inducing hypersensing mutant cells together with a marker gene at low densities within a healthy tissue could test whether immune surveillance acts to remove these cells; the mutant cells should be removed in wild-type backgrounds but proliferate in T cell deficient backgrounds. ASHM can be further tested by seeking, in healthy organisms, autoreactive T cells against the secretion pathway proteins among tissue-resident T cells of the target organ or in adjacent lymph nodes (Weisberg et al., 2019). These T cells should be active and kill cells in the healthy tissue. The killed cells should be preferentially those with high secretion. These experiments are feasible in principle. Another approach would be to test whether people with HLA variants that are protective for autoimmune disease have increased prevalence of hypersecreting adenomas. Identifying the molecular pathways for ASHM, and the nature of the participating T cells, may provide targets to tune its strength (the parameterγ) by pharmacological means, allowing control of the tradeoff between mutant expansion and autoimmune disease.

## STAR★Methods

### Key Resources Table

**Table:** 

REAGENT or RESOURCE	SOURCE	IDENTIFIER
**Deposited Data**		

Curated data of autoreactive TCR beta chain CDR3 sequences	This paper	Table S4
Mouse public TCR beta chain CDR3 sequences	Madi et al., 2014	Table S5 (This paper), SRA: SRP042610
Human public TCR beta chain CDR3 sequences	ADAPTIVE immuneACCESS database	Table S5 (This paper), https://doi.org/10.21417/B7001Z

**Software and Algorithms**		

Wolfram Mathematica version 11.3	Wolfram	https://www.wolfram.com/mathematica/
Simulations of ASHM model	This paper	https://github.com/yaelkorem-weizmann/ASHM_dynamics

### Resource Availability

#### Lead Contact

Further information and requests for resources should be directed to and will be fulfilled by the Lead Contact, Uri Alon (uri.alon@weizmann.ac.il).

#### Materials Availability

Further information and requests for resources should be directed to and will be fulfilled by the Lead Contact, Uri Alon (uri.alon@weizmann.ac.il).

#### Data and Code Availability

The published article includes all datasets generated or analyzed during this study. Simulation code is available at GitHub [https://github.com/yaelkorem-weizmann/ASHM_dynamics].

### Method Details

#### Public TCR analysis

TCR beta chain sequences (CDR3 region) of T cells experimentally found to react with disease autoantigens were retrieved by literature search (Bagaev et al., 2020, Culina et al., 2018, Gomez-Tourino et al., 2017, Lang et al., 2001, Li et al., 2010, Matsuoka et al., 1994, Tickotsky et al., 2017, Trautmann et al., 2002, Zarour et al., 1996). A total of 1000 sequences was thus collected (Table S4). The public TCR beta chains (Table S5) were obtained from public-TCR databases as follows. A mouse database was retrieved from (Madi et al., 2014), using TCR sequences present in at least 25 of 28 mice studied (1005 sequences). A human public repertoire database, was built using the ADAPTIVE immuneACCESS database (Emerson et al., 2017), using sequences shared by at least 400 people out of 785 (5016 sequences). We sought perfect sequence matches to the public TCR databases (perfect matches are listed in Table S3). There were 92 sequences that appeared in both mice and human public repertoire datasets. Perfect matches found in this restricted list are underlined in Table S3.

A statistical analysis for the likelihood of such perfect matches is not relevant for the present study, because the biological question is whether self-reactive TCRs are present in healthy individuals.

#### Mathematical model for ASHM

Consider a cell clone $x_{i}$ that has a net growth rate, proliferation minus removal, that rises with the perceived signal $u_{i}s$, $f\left(u_{i}s\right)$, and a secretion rate that also rises with the perceived signal $g\left(u_{i}s\right)$ (Figure 3A). ASHM removes cells at a rate that rises with antigen level, which is proportional to the cells secretion rate g, relative to the average level of antigen in the tissue $\bar{g}=\sum x_{i}g\left(u_{i}s\right)/\sum x_{i}$. The normalization by average antigen level accounts for the effect of regulatory T cells, that are activated by antigens collected by antigen presenting cells from the tissue, and inhibit cytotoxic T cells (Arias et al., 2015, Grossman and Paul, 1992, Sontag, 2017). Other tolerance mechanisms such as checkpoint inhibition of T cell action might provide similar effects. Hence, ASHM removal goes as $h\left(g\left(u_{i}s\right)/\bar{g}\right)$. Thus,

(Equation 1) $$\frac{dx_{i}}{dt}=x_{i}\left(f\left(u_{i}s\right)-h\left(g\left(u_{i}s\right)/\bar{g}\right)\right)=x_{i}\mu \left(u_{i}s\right)$$

#### Conditions for ASHM mutant removal

To find conditions in which ASHM can remove any mis-sensing mutant (any $u_{i}$) on a background of wild-type cells $\left(u_{i}=1\right)$, we assume that signal is at its homeostatic set-point $s^{*}$, and require that the wild-type growth rate $\mu \left(s^{*}\right)$ is larger than that of any mutant $\mu \left(u_{i}s^{*}\right)$. Mathematically, we demand that at $u_{i}=\bar{u}=1$: (i) (dx/dt)=0 (ii) $\left(d\mu /du_{i}\right)=0$ and (iii) $\left(d^{2}\mu /du_{i}^{2}\right)<0$. If we further assume that the proliferation and antigen secretion are governed by the signal through the same signaling pathway and hence depend on the signal in the same way, i.e., that $f\left(u_{i}s\right)=g\left(u_{i}s\right)-s_{0}$ (where $s_{0}$ is the natural removal rate of the tissue), these requirements translate into constraints on the shape of the ASHM removal function $h\left(g\left(u_{i}s\right)/\bar{g}\right)$:

(Equation 2) $$h^{\text{'}}\left(1\right)=s_{0}+h\left(1\right)\;\;\left(\text{from}\;\left(\text{i}\right)\;\;\text{and}\;\left(\text{ii}\right)\right)\text{,}\;\text{and}$$

(Equation 3) $$h^{''}\left(1\right)>0\;\left(\text{from}\;\left(\text{iii}\right)\right)$$

Notably, these conditions do not depend on the functions *f* and *g*. For ASHM to be an evolutionary stable strategy (Smith and Price, 1973), the conditions suggest that ASHM killing *h* needs to rise quickly near the steady-state point – i.e., to differentially sense hyper-secretors from wild-type cells.

#### ASHM model with linearized functions

For linearized secretion and growth functions, $f=\mu_{0}\left(u_{i}\;s-s_{0}\right)$, $g=g_{0}u_{i}s$, and Hill-like killing with cooperativity *n*, $h=a{\left(g\left(u_{i}s\right)/\bar{g}\right)}^{n}$, Equation (1) becomes:

(Equation 4) $$\frac{dx_{i}}{dt}=x_{i}\left(\mu_{0}\left(u_{i}\;s-s_{0}\right)\;-a{\left(\frac{u_{i}}{\bar{u}}\right)}^{n}\right)$$

In this case, Equation (2) on h yields the relation γ=1/(n−1), where γis the “ASHM strength” parameter, defined as the removal rate of wild-type cells by ASHM in units of their natural removal rate, $\gamma =a/\mu_{0}s_{0}$. Thus, for T cell cooperativity n = 5, the required ASHM strength is small, γ=1/4. This means that ASHM can kill at about 25% of the natural removal rate and still be effective at removing mutants with any value of u. In the case of Hill-like killing with cooperativity n, Equation (3) gives n>2. This means that ASHM needs to be cooperative.

#### Simulation equations and parameters

To model the effect of mutant expansion on homeostasis, we use the model of Karin et al. (Karin et al., 2016), namely that the signal *s* is produced at rate *p* and removed at a rate that is enhanced by the secreted molecule *y.* Thus, ds/dt=p−rys. The molecule y is secreted by the different clones $dy/dt=b\sum x_{i}g\left(u_{i}s\right)-\alpha \;y$. We use linear secretion and growth functions and Hill-like ASHM killing as in Methods section *Conditions for ASHM mutant removal*. We rescale time to tissue turnover rates $t\to \mu_{0}s_{0}t$ and define the rescaled ASHM strength parameter as $\gamma =a/\mu_{0}s_{0}$. The resulting equation is

(Equation 5) $$dx_{i}/dt=x_{i}\left(u_{i}\frac{s}{s_{0}}\;-1-\gamma {\left(\frac{u_{i}}{\bar{u}}\right)}^{n}\right)$$

The wild-type steady-state set-point is $s^{*}=s_{0}\left(1+\gamma \right)$, $x^{*}=\left(p\alpha /rb\right)/s_{0}^{2}{\left(1+\gamma \right)}^{2}$, $y^{*}=\left(p/rs_{0}\right)/\left(1+\gamma \right)$. Thus the ASHM strength γ affects the set-point of the signal and cells. In autoimmune disease (large γ), secretory tissue $x^{*}$ is reduced and the signal $s^{*}$ loses homeostasis (Figures 3J–3L).

When simulating the model, we used the fact that the dynamics of molecule *y* secretion and signal *s* inhibition are typically much faster than the dynamics of tissue turnover. We thus assumed separation of timescales in which signal dynamics is much faster than cell growth, and used a quasi-steady-state approximation for the dy/dt and ds/dt equations. We rescaled *p* such that $\hat{p}=\left(\;p\alpha /brs_{0}^{2}\right)$. Cell concentrations were normalized to the steady-state cell concentration. The resulting equation is Equation (5) with $\frac{s}{s_{0}}=\sqrt{\hat{p}/\sum u_{i}x_{i}}$. The negative feedback on the signal s sets an effective carrying capacity for the mutant and the wild-type populations. In Figure 3C, we defined the autoimmune disease region where there is tissue loss of > 50%. The mutant expansion region was defined as the range of parameters in which a single mutant cell can invade the wild-type (u=1) steady state (i.e., the steady-state becomes an unstable saddle). We used a mutant with u=2. In Figures 3E, 3F, 3H, 3I, 3K, and 3L, we simulated competition between a wild-type and two mutant populations of hyper- and hypo- secretors (u=1 versus u=2 and u=0.5). The mutant initial concentrations were half that of the wild-type cells, and parameters wereγ=0.01,0.25,2 and n=5. In Fig. d,g,j (phase portraits) we simulated a competition between wild-type and hypersecreting mutant only (u = 1 versus u = 2 with γ=0.01,0.25,2 and n=5). All simulations were performed using Wolfram Mathematica 11.3.

#### Regulatory circuits for cell secretion and growth

Here we provide details about the tissue feedback circuits that appear in Figures 1C and 2A.

##### Beta cells

Pancreatic beta cells secrete insulin, the main regulator of blood glucose. Insulin acts to reduce blood glucose by inducing uptake by peripheral tissue and reducing glucose production by the liver. An increase in blood glucose concentration stimulates the secretion of insulin by pancreatic beta cells. In addition, glucose increases beta-cell growth in rodents and humans (Levitt et al., 2011, Stamateris et al., 2016). At very high levels, glucose causes beta cell functional decline and death, in a process called glucotoxicity. This biphasic effect of glucose can act to protect against strong mis-sensing mutants (Karin and Alon, 2017).

##### Thyroid gland

Thyroid gland follicular cells secrete thyroid hormones T4 and T3 in response to thyroid stimulating hormone (TSH) secreted by the pituitary thyrotropes. T4 is converted into T3 in peripheral tissue cells, controlling many aspects of metabolism. T4 and T3 in turn suppress TSH secretion, forming a negative feedback that keeps thyroid hormone concentration at homeostasis (Berberich et al., 2018). TSH is also the major growth factor for thyroid gland cells (Dumont et al., 1992, Rivas and Santisteban, 2003). Thyroid growth provides a long-term compensation mechanism that can result in thyroid goiter formation, for example, as in the case of iodine deficiency.

##### Adrenal gland

The adrenal cortex cells found in the zona fasciculata secrete cortisol in response to ACTH stimulation, secreted by the pituitary corticotrophs. Cortisol suppresses ACTH secretion creating a negative feedback loop which regulates stress response (Smith and Vale, 2006). ACTH is the main growth factor of adrenal cortex cells (Hoeflich and Bielohuby, 2009).

##### Skin melanocytes

Skin melanocytes produce melanin, a pigment that protects against UV radiation, and transfer the pigment granules to surrounding keratinocytes. This process is regulated by melanocortins, including melanocyte stimulating hormone (MSH), secreted by the pituitary gland and skin, and ACTH (Slominski et al., 2004). The negative feedback in this circuit is indirect: UVB radiation increases MSH receptor activity and MSH/ACTH precursor production (Chakraborty et al., 1995, Chakraborty et al., 1999). Melanin absorbs UVB radiation, and therefore reduces MSH and ACTH levels and activity. MSH promotes melanocyte proliferation and survival (Kadekaro et al., 2003).

##### Stomach parietal cells

These cells secrete acid into the stomach under control of the hormone gastrin. Gastrin is secreted by stomach cells when pH is not acidic enough. Gastrin is the main growth factor for stomach parietal cells (Dockray, 1999).

##### Liver cholangiocytes

Cholangiocytes line the bile ducts in the liver. They secrete water and bicarbonate under control of the hormone secretin. This hormone is secreted by cells in the duodenum when pH is acidic. Secretin production is inhibited by bicarbonate in the bile which reduces acidity in the duodenum. Secretin is the main growth factor for cholangiocytes (Afroze et al., 2013, Munshi et al., 2011).

##### Renin secreting cells

The juxtaglomerular cells in the kidney secrete renin that regulates blood pressure through a hormone cascade including angiotensin. Renin secretion by juxtaglomerular cells is inhibited by high blood pressure (Harrison-Bernard, 2009). High blood pressure was also suggested to inhibit the proliferation of these cells (Owen et al., 1995, Turgeon and Sommers, 1961). For example, in “Goldblatt kidney” patients, the adjacent blood vessels are partially blocked, causing the juxtaglomerular cells to sense a locally low blood flow. This results in increased renin secretion leading to high systemic blood pressure (Navar et al., 1998). Turgeon and Sommers found hyperplasia of juxtaglomerular cells in these patients, which was inversely correlated with hypertension duration.

##### Pituitary corticotophs

Corticotroph cells secrete ACTH and other POMC peptides, when activated by CRH secreted by the hypothalamus. CRH is the main growth factor for corticotrophs (Gertz et al., 1987, Nolan et al., 1998). ACTH inhibits CRH by an indirect path: ACTH activates the adrenal cortex to secrete cortisol, which shuts down CRH (and ACTH) production (Smith and Vale, 2006). There is also evidence for a direct inhibition of CRH secretion by ACTH in what is called a short feedback loop (Papadimitriou and Priftis, 2009).

##### Pituitary somatotrophs

Somatotroph cells secrete growth hormone (GH) when activated by GHRH secreted by the hypothalamus (Camacho-Hübner et al., 2000). GHRH is the main growth factor for somatotrophs (Billestrup et al., 1986). GH inhibits GHRH secretion by an indirect path: GH activates the liver to secrete IGF1, which shuts down GHRH (and GH) production (Camacho-Hübner et al., 2000).

##### Pancreatic alpha cells

Alpha cells in the pancreas secrete the hormone glucagon, which activates glucose production in the liver. The regulation of secretion and proliferation in these cells is complex and a subject of current research. Glucagon secretion is inhibited by glucose, and glucagon is a growth factor for alpha cells (Liu et al., 2011). If glucagon acts in a local autocrine manner, this circuit is prone to mutant expansion of glucagon hyper-secreting cells, including glucose hypo-sensing alpha cells. Such cells secrete more glucagon and due to the autocrine effect, have higher growth rate. There are additional regulatory steps making the circuit more complex, and prohibiting a decisive analysis at this stage. For example, insulin also plays a role in glucagon secretion and alpha cell proliferation (Garzilli and Itzkovitz, 2018).

#### Estimates of the fitness costs of hyper- and hyposecretion

In order to estimate the fitness cost of hyper/hypo secretion in Figure 2D, we use as a proxy the physiological concentration range of each secreted factor. We tabulated three parameters: 1. The normal concentration level, 2. The concentration under which there is acute life danger 3. The concentration above which there is an acute life danger. We did not consider long-term harmful effects of chronic unbalanced factor levels, because acute lethality carries a higher fitness cost than morbidity at old ages. As a first approximation for the severity of the effect of mild hyper/hypo secretion respectively, we computed the ratios between the highest and normal concentrations as $F_{hyper}$, and the normal to lowest concentrations $F_{hypo}$, repsectively. In Figure 2D we show $\text{log}\left(F_{hypo}/F_{hyper}\right)$. Positive values indicate that the system is more sensitive to hypo-secretion than to hyper-secretion. We found estimates for six of the relevant factors in this study, as follows. PTH: we use ionized calcium level as a proxy. Normal levels are around 1.2 mM, severe hypocalcemia occurs below 0.9 mM (Steele et al., 2013). Hypercalcemic crisis occurs above 2.5 mM (Carroll and Schade, 2003). Insulin: we use glucose levels as a proxy. Normal glucose concentration is 5 Mm, while lethal low concentration leading to neuroglycopenic symptoms is 3 mM (Cryer, 2007). High concentrations of glucose can lead to hyperosmolar crisis, characterized by glucose levels higher than 14 mM. Additionally, low insulin concentrations may result in unlimited lipolysis leading to ketoacidosis (Westerberg, 2013). Glucagon: we use glucose levels as a proxy, with the same normal and lethal-low concentrations as for insulin. However, the main lethal effect of high glucagon is not ketoacidosis but hyperglycemic hyperosmolar syndrome, which happens above 33 mM glucose. Renin: we use systolic blood pressure as a proxy. Normal level is 120 mmHg. Hypotension occurs below 90 mmHg and can be lethal because of fainting or shock. Hypertensive crisis can occur above 180 mmHg (values from the US National Heart, Lung, And Blood Institute). Considering diastolic blood pressure instead gives the same results. Thyroxine: we use free T4 concentrations. Normal concentration is around 1.3 ng/dl. Lethal high value is 10.9 ng/dl (Thyroid storm, (Brooks and Waldstein, 1980)). Lethal low levels occur around 0.1 ng/dl (danger of myxedema coma, estimation based on case reports, e.g., (Charoensri et al., 2017), and physiological distribution of FT4 levels in (Hoermann et al., 2010)). Gastric acid: we use stomach pH levels. Normal baseline level is around 1.4 (McLauchlan et al., 1989). We took low lethal level to be beneath pH of 1, which can lead to a potentially life threatening peptic ulcer (Pizzorno et al., 2016). We could not find values for high lethal levels, for a conservative estimate we took the highest physiological level, pH 7, which can occur after a meal (McLauchlan et al., 1989).

#### The probability of hypersecreting adenomas per cell is much higher in the pituitary than in the adrenal gland

The ASHM hypothesis predicts a tradeoff between organ-specific autoimmune disease and hyper-secreting mutant expansion diseases. One example is the case of pituitary gland corticotrophs that secrete ACTH versus the adrenal cortx cells that secrete cortisol. Hyper-secreting mutants in both organs result in adenomas that cause a similar syndrome – pathologically high cortisol levels called Cushing’s syndrome. ACTH-hyper-secreting adenomas in the pituitary gland, which is less prone to autoimmune disease, are much more common per cell than hypersecreting adenomas in the adrenal cortex. To see this, note that the pituitary gland weighs ∼0.5g, and corticotrophs make up 15%–20% of the anterior pituitary cells (Amar and Weiss, 2003). The two adrenal glands weigh ∼10 g together, of which ∼50% are cortisol-secreting cells (Neville and O’Hare, 2012; Wallig et al., 2017). Thus there are about 50 times more of the relevant cells in the adrenal. However, about 80% of Cushing’s syndrome cases are caused by pituitary tumors, and only 20% are from adrenal tumors (Castinetti et al., 2012). This means that per cell, the chance for a hyper-secreting tumor of pituitary corticotrophs is about 250 times larger than in adrenal cortisol secreting cells. This agrees with the predicted tradeoff.

We note that ASHM theory does not preclude tissues with common autoimmune disease to also have relatively prevalent hypersecreting mutant expansion diseases. The ASHM theory suggests that if ASHM were absent, the prevalence of such mutant expansion disease would be much higher. An example is thyroid toxic nodules that hypersecrete thyroid hormone, which have a prevalence on the order of 1%, and occur in an organ with frequent autoimmune disease (Hashimoto’s thyroiditis). Other tissues with common autoimmune disease have extremely rare diseases of mutant hyper-expansion, such as insulinomas (Table S1).

#### Remark on ASHM and cancer

The hyper-secreting mutant diseases we discuss are typically non-cancerous. It is noteworthy that thyroid carcinomas (as opposed to thyroid toxic nodules which are not cancerous) rarely secrete their cognate hormone thyroxin (Greenspan, 1974), perhaps due in part to the need to evade ASHM. On the other hand, in some cases when autoimmunity and cancer co-occur in the same tissue, the cancer tends to be less aggressive and patients have better prognosis. This was shown for melanoma and vitiligo (Byrne and Turk, 2011, Bystryn et al., 1987, Nordlund et al., 1983). Thus ASHM may serve as a barrier to cancer development in some tissues.

#### Remark on female prevalence of autoimmune disease

The ASHM model can offer a putative explanation for why most of the common autoimmune disease are more prevalent in females. Endocrine organs such as thyroid and adrenal show more turnover during pregnancy, and thus run the risk for more mutations. This may require stronger ASHM against these organs in females. One prediction is that endocrine organs that do not show autoimmune disease but proliferate in pregnancy should show more hypersecreting mutant disease in females than in males. This is indeed the case for primary hyperparathyroidism (female:male of 3:1) (Adami et al., 2002) and pituitary adenomas (ACTH secreting 5:2, PRL secreting 5:2) (Mindermann and Wilson, 1994). The exceptions are illuminating: T1D has about 1:1 F:M bias (Cooper and Stroehla, 2003), and so do TSH-secreting pituitary adenomas (Mindermann and Wilson, 1994). Growth-hormone secreting pituitary adenomas are more frequent in males (2:5) (Mindermann and Wilson, 1994). These three systems correspond to sexual dimorphism of larger size in males and potentially more cell divisions; vitiligo shows a 1:1 bias, however pregnancy is a known trigger for vitiligo disease onset (Cooper and Stroehla, 2003, Driessche and Silverberg, 2018).

Postpartum there are prevalent flares of autoimmune thyroiditis, often transient (Stagnaro-Green, 2002). These are surprising because autoimmunity is often absent before pregnancy. ASHM suggests that due to lowered adaptive immunity during pregnancy (Stagnaro-Green et al., 1992), mutant cells can expand without being removed. This might cause the increase in thyroid nodule number and size during pregnancy (Kung et al., 2002). Postpartum, when adaptive immunity is restored, ASHM attacks the mutant clones causing thyroiditis. The transient nature of the phenomenon might reflect a successful removal of these mutant clones.

## Supplemental Information

Document S1. Tables S1–S3

Table S4. Annotated Autoreactive TCR Beta Chain CDR3 Sequences, Related to STAR Methods

Table S5. Public Repertoire TCR Beta Chain CDR3 Sequences for Human and Mouse, Related to STAR Methods

Document S2. Article plus Supplemental Information
