# Supplementary material for: Endocrine Autoimmune Disease as a Fragility of Immune Surveillance against Hypersecreting Mutants
Source: Immunity. 2020 May 19;52(5):872–884.e5. doi: 10.1016/j.immuni.2020.04.022 (PMC7237888; doi:10.1016/j.immuni.2020.04.022)
Supplement: Document S1. Tables S1–S3 [file mmc1.pdf]

Immunity, Volume *52*

Supplemental Information

Endocrine Autoimmune Disease as a Fragility of Immune Surveillance against Hypersecreting Mutants

Yael Korem Kohanim, Avichai Tendler, Avi Mayo, Nir Friedman, and Uri Alon

Supplemental Information

Tables S1-S3

Table S1: Endocrine autoimmune diseases. Related to Figure 1.

Autoimmune Disease	Cells	Prevalence of AID	Input	Secreted factor	Auto-antigens	Role of autoantigen*	Prevalence of disease of hypersecreting mutant expansion
Type-1 Diabetes	Pancreatic beta cells	0.1-1% (Cooper and Stroehla, 2003; Cooper et al., 2009)	glucose	Insulin	Insulin	Insulin	Insulinoma, ~0.01% (Service et al., 1991)
					Preproinsulin (PPI)	Insulin precursor	
					Protein Tyrosine Phosphatase, Receptor Type N (PTPRN)	Insulin storage and secretion (Harashima et al., 2012; Saeki et al., 2002)	
					Protein Tyrosine Phosphatase, Receptor Type N2 (PTPRN2)	Insulin secretion regulation (Cai et al., 2011; Doi et al., 2006)	
					Islet cell antigen-69 (ICA69)	transport of insulin secretory granules (Buffa et al., 2008)	
					Zinc transporter 8 (ZnT8)	Insulin production, storage, and secretion (Chimienti et al., 2006; Davidson et al., 2014)	
					Glutamic decarboxylase 65 (GAD65)	No known role in insulin secretion	
Thyroiditis (Hashimoto's disease)	Thyroid gland cells (thyrocytes)	0.1-1% (Cooper and Stroehla, 2003; Cooper et al., 2009)	TSH	T4/ T3	Thyroid peroxidase (TPO)	T3/T4 biosynthesis (Ruf and Carayon, 2006)	Thyroid toxic adenoma (toxic nodule), ~0.5%-1% (Bürgi et al., 1998)
					Thyroglobulin (Tg)	T3/T4 precursor	
Addison's Disease	Adrenal cortex cells	~0.01% (Cooper and Stroehla, 2003; Cooper et al., 2009)	ACTH	Cortisol/ aldosterone	21-hydroxylase (CYP21A2)	Cortisol/ aldosterone biosynthesis	Adrenal Cushing's syndrome, ~ 0.0008% (Castinetti et al., 2012)

Vitiligo	Melanocytes	0.1-1% (Cooper and Stroehla, 2003; Cooper et al., 2009; Krüger and Schallreuter, 2012)	MSH	Melanin	Melanocyte protein PMEL	Melanin synthesis and storage (Raposo and Marks, 2007)	?
					Protein melan-A (MART1)		
					Tyrosinase (TYR)	Melanin synthesis (Lai et al., 2018)	
					Tyrosinase related proteins 1 and 2		
Autoimmune gastritis	Stomach parietal cells	0.1-1% (Cooper and Stroehla, 2003; Cooper et al., 2009; Kulnigg-Dabsch, 2016; Minalyan et al., 2017)	Gastrin	Acid	Gastric H/K ATPase	Acid production (Kulnigg-Dabsch, 2016)	?
Primary biliary cirhosis	Cholnagiocytes	~0.01% (Cooper and Stroehla, 2003; Cooper et al., 2009)	Secretin	Bicarbonate	PDC-E2 ,E3BP components of pyruvate/oxo-glutarate dehydrogenase	Bicarbonate production (CO2)	?

*Autoantigens are from: T1D: (Roep and Peakman, 2012) – T cell antigens. Hashimoto thyroiditis: (Chistiakov, 2005) – autoantibodies with evidence for T cell reactivity. Addison Disease: (Michels and Eisenbarth, 2010) – autoantibodies. Vitligo (Richmond et al., 2013) – T cell antigens. Autoimmune gastritis: (Kulnigg-Dabsch, 2016), T cells antigen. PBC: (Lleo et al., 2014) – autoantibodies. ?- no clear corresponding disease.

Table S2: Tissues that rarely get autoimmune diseases. Related to Figure 2.

**Cells**	**Input**	**Secreted factor**	**Autoimmune Disease**	**Prevalence of autoimmune disease**	**Hypersecreting mutant expansion disease**	**Prevalence of hypersecreting mutant expansion disease**
Parathyroid gland cells	Calcium	PTH	Autoimmune hypoparathyroidism	10^{-3}-10^{-4}% (Mostly as a part of autoimmune polyendocrine syndrome type 1)	Primary hyperparathyroidism	0.1-1% (Yeh et al., 2013)
Alpha cells	Insulin	Glucagon	No known disease	**-**	Hyperglucagonemia	?
Kidney juxtaglomerular cells	Blood pressure	Renin	No known disease	**-**	Renin-secreting juxtaglomerular cell tumor/ reninoma	~0.01%*****
Corticotrophs	CRH	ACTH	Autoimmune hypophysitis	0.0005% (Howlett et al., 2010)	ACTH secreting adenoma	1%**
Somatotrophs	GHRH	GH			GH secreting adenoma	0.6%**
Thyrotrophs	TRH	TSH			TSH secreting adenoma	0.1%**
Lactotrophs	Dopamine, TRH	Prolactin			Prolactin secreting adenoma	9%**
Gonadotrophs	GnRH	FSH, LH			Gonadotropin secreting adenoma	0.3%**

* Renin-secreting juxtaglomerular cell tumors are a rare cause for hypertension (Kuroda et al., 2011). Haab et al found that 8 out of 30,000 hypertensive patients at a hypertensive clinic had juxtaglomerular cell tumors (Haab et al., 1995). The prevalence of hypertension in the population is 29% ((Fryar et al., 2017), in the US). We thus roughly estimate the prevalence of juxtaglomerular cell tumors as 0.29×8/30,000.
** Pituitary hormone secreting adenoma prevalence was calculated as follows: pituitary adenoma prevalence in the population is ~20% (Ezzat et al., 2004). McComb et al estimated on the basis of 107 studied adenomas that ~50% of the adenomas are hormone secreting (McComb et al., 1983), the vast majority of which are sub-clinical. About 43% secrete prolactin, 2.8% secrete GH, 4.9% secrete ACTH, 1.4% secrete LH, and 0.7% secrete TSH (Ezzat et al., 2004; McComb et al., 1983).

Table S3: Autoimmune TCR β-chain CDR3 sequences are found in the public TCR repertoire of healthy individuals. Related to Methods. Comparison between i. curated TCR sequences that recognize antigens identified in autoimmune diseases (Table S4, sequences were taken from the indicated references) and ii. Public TCR repertoire datasets that contain sequences that frequently appear in mice (for thyroglobulin) or in human (for the rest) (Table S5). Autoimmune TCR sequences that appear in the public repertoire dataset are listed here. Sequences that are public in both human and mouse are underlined. (Methods).

**Autoimmune disease**	**Antigen**	**TCR β-chain CDR3 sequences in public dataset**	**References**
Hashimoto's thyroiditis	Thyroglobulin	CASSLGEQYF<br>CASSRDSSQNTLYF<br>CASSRDSYAEQFF<br>CASSRTNTEVFF	(Matsuoka et al., 1994)
T1D	ZNT8	CASSDQETQYF	(Culina et al., 2018)
	Proinsulin	CASSLERETQYF	(Gomez-Tourino et al., 2017)
	Islet autoreactive	CASSLVGGNEQFF	
	GAD65	CASSELYEQYF CASSERETQYF<br>CASSLATYEQYF CASSLGDQPQHF<br>CASSLGGYGYTF<br>CASSLGLTDTQYF CASSLQYEQYF<br>CASSLRAGELFF<br>CASSLTGELFF CASSPGGGYTF<br>CASSPTGYEQYF CASSQGDYGYTF<br>CASSQGGGNQPQHF<br>CASSRQGTGELFF<br>CASSRTGYGYTF<br>CASSSGTVNTEAFF<br>CASSFSSGNTIYF<br>CASSLAMNTEAFF CASSLEGGYTF<br>CASSLGTSSYEQYF<br>CASSLQGTYEQYF<br>CASSLVGGNEQFF<br>CASSPGQGNTIYF<br>CASSPGTAGNTIYF<br>CASSQTDTQYF CASSRGTEAFF	
Vitiligo	PMEL (gp100)	CASSLGSSYEQYF	The TCR sequence is from (Zarour et al., 1996). It recognizes the epitope YLEPGPVTA, which was found to be an auto-epitope in a vitiligo patient (Lang et al., 2001).
	MART1 (MLANA)	CASSNSYEQYF, CASSLGDTEAFF	TCR sequences are from (Trautmann et al., 2002), through the VDJdb (Bagaev et al., 2020). These sequences are against the epitope ELAGIGILTV that was recognized as an autoantigen in vitiligo (Lang et al., 2001; Li et al., 2010)
